# Hepatitis B testing, treatment, and virologic suppression in HIV-infected patients in Cameroon (ANRS 12288 EVOLCAM)

**DOI:** 10.1186/s12879-020-4784-7

**Published:** 2020-01-15

**Authors:** Florian Liégeois, Sylvie Boyer, Sabrina Eymard-Duvernay, Patrizia Carrieri, Charles Kouanfack, Jenny Domyeum, Gwenaëlle Maradan, Jacques Ducos, Eitel Mpoudi-Ngolé, Bruno Spire, Eric Delaporte, Christopher Kuaban, Laurent Vidal, Christian Laurent, for the EVOLCAM study group, C. Kuaban, L. Vidal, G. Maradan, A. Ambani, O. Ndalle, P. Momo, C. Tong, S. Boyer, V. Boyer, P. J. Coulaud, L. March, M. Mora, L. Sagaon‐Teyssier, M. de Sèze, B. Spire, M. Suzan‐Monti, C. Laurent, F. Liégeois, E. Delaporte, S. Eymard‐Duvernay, Adeline Riondel, F. Chabrol, E. Kouakam, O. Ossanga, H. Essama Owona, C. Biloa, M.‐. T. Mengue, E. Mpoudi‐Ngolé, P. J. Fouda, C. Kouanfack, H. Abessolo, N. Noumssi, M. Defo, H. Meli, Z. Nanga, Y. Perfura, M. Ngo Tonye, O. Kouambo, U. Olinga, E. Soh, C. Ejangue, E. Njom Nlend, A. Simo Ndongo, E. Abeng Mbozo’o, M. Mpoudi Ngole, N. Manga, C. Danwe, L. Ayangma, B. Taman, E. C. Njitoyap Ndam, B. Fangam Molu, J. Meli, H. Hadja, J. Lindou, J. M. Bob Oyono, S. Beke, D. Eloundou, G. Touko, J. J. Ze, M. Fokoua, L. Ngum, C. Ewolo, C. Bondze, J. D. Ngan Bilong, D. S. Maninzou, A. Nono Toche, M. Tsoungi Akoa, P. Ateba, S. Abia, A. Guterrez, R. Garcia, P. Thumerel, E. Belley Priso, Y. Mapoure, A. Malongue, A. P. Meledie Ndjong, B. Mbatchou, J. Hachu, S. Ngwane, J. Dissongo, M. Mbangue, Ida Penda, H. Mossi, G. Tchatchoua, Yoyo Ngongang, C. Nouboue, I. Wandji, L. Ndalle, J. Djene, M. J. Gomez, A. Mafuta, M. Mgantcha, E. H. Moby, M. C. Kuitcheu, A. L. Mawe, Ngam Engonwei, L. J. Bitang, M. Ndam, R. B. Pallawo, Issiakou Adamou, G. Temgoua, C. Ndjie Essaga, C. Tchimou, A. Yeffou, I. Ngo, H. Fokam, H. Nyemb, L. R. Njock, S. Omgnesseck, E. Kamto, B. Takou, L. J‐. G. Buffeteau, F. Ndoumbe, J‐. D. Noah, I. Seyep

**Affiliations:** 1 Institut de Recherche pour le Développemen, Inserm, Univ Montpellier, TransVIHMI, 911 avenue Agropolis, BP 64501, 34394 Montpellier, cedex 5 France; 2 CREMER, Yaoundé, Cameroon; 3 Present Address: IRD, CNRS, Univ Montpellier, MIVEGEC, Montpellier, France; 4 Inserm, IRD, Univ Aix-Marseille, SESSTIM, Marseille, France; 5 Central Hospital, Yaoundé, Cameroon; 6 Faculty of Medicine and Pharmaceutical Sciences, University of Dschang, Dschang, Cameroon; 7 Laboratory of Viral Hepatitis, Inserm, University Hospital, Montpellier, France; 8 Faculty of Medicine and Biomedical Sciences, University of Yaoundé I, Yaoundé, Cameroon

**Keywords:** Africa, HBV, HIV, Testing, Treatment

## Abstract

**Background:**

Hepatitis B is a major concern in Africa, especially in HIV-infected patients. Unfortunately, access to hepatitis B virus (HBV) testing and adequate treatment remains a challenge in the continent. We investigated HBV testing, treatment, and virologic suppression in HIV-infected patients followed up as part of Cameroon’s national antiretroviral programme.

**Methods:**

A cross-sectional survey was performed in adult patients receiving antiretroviral therapy (ART) in 19 hospitals in the Centre and Littoral regions in Cameroon. The proportions of patients tested for hepatitis B surface antigen (HBsAg) prior to the study were compared among all study hospitals using the Chi-square test. The association of individual and hospital-related characteristics with HBV testing and virologic suppression was assessed using multilevel logistic regression models.

**Results:**

Of 1706 patients (women 74%, median age 42 years, median time on ART 3.9 years), 302 (17.7%) had been tested for HBsAg prior to the study. The proportion of HBV-tested patients ranged from 0.8 to 72.5% according to the individual hospital (*p* < 0.001). HBV testing was lower in women (adjusted odds ratio [aOR] 0.64, 95% confidence interval [CI] 0.46–0.89, *p* = 0.010) and higher in patients who initiated ART in 2010 or later (aOR 1.66, 95% CI 1.23–2.27, *p* < 0.001). Of 159 HBsAg-positive patients at the time of the study (9.3%), only 97 (61.0%) received Tenofovir + Lamivudine (or Emtricitabine). Of 157 coinfected patients, 114 (72.6%) had a HBV viral load < 10 IU/mL. HBV suppression was higher in patients with a HIV viral load < 300 copies/mL (aOR 3.46, 95% CI 1.48–8.09, *p =* 0.004) and lower in patients with increased ALT level (aOR 0.86 per 10 IU/mL increase, 95% CI 0.75–0.97, *p* = 0.019).

**Conclusions:**

A substantial proportion of HIV/HBV coinfected patients were at higher risk of liver disease progression. Improving the management of HBV infection in the routine healthcare setting in Africa is urgently required in order to achieve the 2030 elimination targets. Micro-elimination of HBV infection in people living with HIV could be an easier and cost-effective component than more widely scaling up HBV policies.

## Background

Elimination of viral hepatitis as a major public health threat by 2030 is part of the Sustainable Development Goals. The World Health Organization (WHO) adopted a global strategy in 2016, which aims to reduce the number of new cases of chronic hepatitis B and C by 90% and the number of deaths associated with these diseases by 65% by 2030 [1]. Testing, treatment, and virologic suppression are crucial elements of this strategy.

Hepatitis B is a major concern in Africa, especially in HIV-infected patients who have a greater risk of liver failure, cirrhosis, hepatocellular carcinoma, and death [2]. Of the 36.7 million people living with HIV worldwide in 2015, approximately 2.7 million (7.4%) were coinfected with hepatitis B virus (HBV) [3]. Nearly three quarters of the latter resided in Africa. In a recent meta-analysis, the prevalence of HBV infection in people living with HIV was estimated at 8.4% worldwide and up to 12.4% in West and Central Africa [4].

Since 2010, the WHO has recommended that if feasible, all patients be tested for hepatitis B surface antigen (HBsAg) at the time of HIV diagnosis [5, 6]. It also recommends the concomitant use of two drugs which are active against both HIV and HBV - tenofovir disoproxil fumarate (TDF) plus lamivudine (3TC) or emtricitabine (FTC) - as part of antiretroviral therapy (ART) in coinfected patients. Since 2013, TDF + 3TC (or FTC) has been the nucleos(t)ide reverse transcriptase inhibitor backbone of the preferred first-line regimen for all HIV-infected adolescent and adult patients [7].

Unfortunately, access to HBV testing and adequate treatment for HIV-infected patients remains a challenge in Africa, and data on the therapeutic response in the routine healthcare setting are lacking because HBV DNA level is not monitored. Only a small proportion of HIV-infected patients are tested for HBV because of laboratory limitations (especially in rural settings) and patients’ financial constraints [8]. Until recently, most HIV/HBV coinfected patients received 3TC-based ART without TDF and were likely to develop HBV resistance [9, 10]. TDF + 3TC (or FTC)-based ART is now increasingly used but the WHO has underlined the need for data on the real coverage of this treatment in HIV/HBV coinfected patients [3]. We therefore aimed to describe HBV testing uptake, treatment, and virologic suppression in HIV-infected adult patients followed up in Cameroon’s national antiretroviral programme.

## Methods

### Study setting

In Cameroon, approximately 10% of HIV-infected patients are positive for HBsAg [9, 11–13]. National guidelines recommended HBsAg testing before starting ART in HIV-infected patients who show signs and symptoms of liver disease [14]. Alanine aminotransferase (ALT) and aspartate aminotransferase (AST) levels were requested for all patients. TDF + 3TC (or FTC) is recommended for patients with HIV/HBV coinfection and those with anaemia since 2007 (as an alternative of zidovudine +3TC). This association became available from 2010 onwards for all HIV-infected patients (independently of HBV status) but is the preferred choice since 2015 only (i.e. after the study). All patients switched to TDF + 3TC (or FTC) in 2016, unless they refused.

### Study population

The cross-sectional ANRS 12288 EVOLCAM survey was performed between March and December 2014 in HIV-infected patients followed up in 19 hospitals in the Centre and Littoral regions in Cameroon [15]. Study hospitals included eight primary HIV services (*“centres de traitement agréés”*) and 11 secondary HIV services (*“unités de prise en charge”*). The former are reference centres for HIV management, and are mostly located in national and regional hospitals. They also serve as mentors for secondary HIV services, which are smaller and less experienced and are mostly located in district hospitals [16]. Patient eligibility criteria for the present study were: aged over 21 years (i.e. legally adult), infection with HIV-1 group M, and on ART for more than 6 months. Participants were selected by enrolling the first patient attending the medical consultation as soon as an interviewer was available. Participants underwent a clinical examination, venous puncture, and provided various data. Data on HIV and HBV clinical, biological and therapeutic histories were collected from routine medical records and a patient interview and recorded in a specific medical file. Data on socio-demographic and economic characteristics, and HBV risky behaviors were recorded using a face-to-face questionnaire performed by trained interviewers. HBV risky behaviors included: number of sexual partners during lifetime, history of incarceration, scarification, tattoo, piercings, use of razor blade or clipper of another person, hospitalization or consultation of a traditional healer in the previous 3 months, and beer consumption. Finally, data on the characteristics of study hospitals were collected from the various services’ medical staff.

### Laboratory procedures

Whole blood samples were collected for screening of HBsAg and measurements of HBV DNA level, ALT and AST levels, HIV-1 RNA level, and CD4 cells count. Plasma samples were tested for HBsAg using a Monolisa HBsAg ULTRA assay (Biorad, Marne la Coquette, France). When the HBsAg result was indeterminate, the sample was retested. When the HBsAg result was positive, the HBV DNA level was quantified using the Abbott RealTime HBV Viral Load assay (Abbott Molecular, IL, USA; sensitivity threshold of 10 IU/mL). HBV genotype was determined in samples with HBV DNA above 250 IU/mL using the INNO-LiPA HBV Genotyping assay (Fujirebio Europe, Gent, Belgium). HIV-1 RNA level was determined using the Generic Viral Load assay (Biocentric, Bandol, France; sensitivity threshold of 300 copies/mL) or the Abbott RealTime HIV-1 quantitative assay (Abbott Molecular, IL, USA; sensitivity threshold of 40 copies/mL). CD4 cell counts were determined using the FACSCount device (Becton Dickinson, Mountain View, CA, USA).

### Statistical analysis

HBV infection was defined as a positive HBsAg test, and HBV suppression as a HBV DNA level below 10 IU/mL. The proportions of patients tested for HBsAg prior to the study were compared among all 19 hospitals using the Chi-square test. The 95% confidence intervals (CI) of the proportions of HBV testing, infection and suppression were computed using the Agresti-Coull method. The association of individual and hospital-related characteristics with HBV testing or suppression was assessed using multilevel logistic regression models, which took into account the correlation between the patients within each hospital by placing the former at the first level and the latter at the second level. Characteristics to be tested were selected a priori on the basis of existing literature about hepatitis B. Those associated with outcomes with a *p*-value below 0.25 in univariate analyses were entered in the complete multivariate models. A manual backward selection was used to determine the final multivariate models. The goodness-of-fit of models was assessed using the Bayesian Information Criterion (BIC). Data were analyzed using Stata 14 software (StataCorp, College Station, Texas, USA).

## Results

### Characteristics of the study population

Of the 1718 patients eligible for the study, 1706 (99.3%) had data for HBsAg and were analyzed. Their characteristics are shown in Table 1. Median time on ART was 3.9 years (interquartile range [IQR] 3.1–4.9). The majority of patients were women (74.0%). Median age was 42 years (IQR 32–52). Three quarters of the patients had a HIV viral load below 300 copies/mL. Median ALT and AST levels were 36 IU/L (IQR 21–42) and 41 IU/L (IQR 28–45), respectively. One hundred and fifty-nine patients were HBsAg positive (9.3, 95% CI 8.0–10.8) according to the results of the tests performed at the time of the present study.

**Table 1:** Characteristics of the study population

Characteristics	N	n (%) or median (IQR)	
Individual characteristics			
Women	1706	1262	(74.0%)
Age (years)	1706	42	(32–52)
Residence	1692		
Urban		1431	(84.6%)
Rural		261	(15.4%)
School educational level	1702		
Never attended school		49	(2.9%)
Primary school		546	(32.1%)
Secondary school		969	(56.9%)
University		138	(8.1%)
Marital status	1680		
Couple		1023	(60.9%)
Single		349	(20.8%)
Divorced or separated		84	(5.0%)
Widowed		224	(13.3%)
Paid activity	1706	1120	(65.7%)
Household monthly income (Franc CFA)^a^	1706	8998	(3333–10,416)
Living below poverty line^b^	1706	1580	(92.6%)
At least 2 meals per day	1706	1245	(73.0%)
HIV clinical stage at ART initiation	1481		
WHO I/II or CDC A/B		601	(40.6%)
WHO III/IV or CDC C		880	(59.4%)
CD4 cell count (cells/mm^3^)	1702	448	(192–704)
HIV viral load < 300 copies/mL	1698	1334	(78.6%)
HBsAg positive	1706	159	(9.3%)
ALT level (IU/L)	1679	36	(21–42)
AST level (IU/L)	1679	41	(28–45)
Body mass index (kg/m^2^)	1668	24.5	(21.3–26.7)
Time since ART initiation (years)	1706	3.9	(3.1–4.9)
Anti-HBV drugs as part of ART	1700		
TDF + 3TC (or FTC)		1028	(60.5%)
3TC (or FTC) alone		582	(34.2%)
TDF alone		40	(2.4%)
None		50	(2.9%)
Adherence to ART in the previous 4 weeks	1699		
Perfect adherence		487	(28.7%)
Adherence difficulties^c^		856	(50.3%)
Treatment interruption for more than 2 consecutive days		356	(21.0%)
Hospitalization in the previous 3 months	1690	144	(8.5%)
Consultation of a traditional healer in the previous 3 months	1699	78	(4.6%)
History of incarceration	1701	44	(2.6%)
Scarification/Tattoo/Piercings	1690	757	(44.8%)
Use of razor blade or clipper of another person	1679	605	(36.1%)
Beer consumption	1702		
Never		757	(44.5%)
Monthly or less		779	(45.7%)
Weekly		147	(8.6%)
Daily		19	(1.1%)
More than 2 h’ journey time to arrive at study hospital	1583	269	(17.0%)
*Characteristics of study hospital attended*			
Region	1706		
Centre		943	(55.3%)
Littoral		763	(44.7%)
Setting	1706		
Urban		991	(58.1%)
Rural		715	(41.9%)
Administrative sector	1706		
Public		1214	(71.2%)
Private		492	(28.8%)
Type of HIV service	1706		
Primary		854	(50.1%)
Secondary		852	(49.9%)
Task-shifting of ART prescription renewals to nurses	1706	1073	(62.9%)
Task-shifting of ART follow-up consultations to nurses	1706	1082	(63.4%)
Stock-out of ART in the previous 12 months^d^	1706	948	(55.6%)

Abbreviations: *ART* antiretroviral therapy, *IQR* interquartile range, *ALT* Alanine aminotransferase, *AST* Aspartate aminotransferase

^a^ 1000 Francs CFA equal approximately 1.5 Euros

^b^ Household monthly income < 28,310 Francs CFA, approximately 43 Euros (Institut National de la Statistique. Quatrième Enquête Camerounaise Auprès des Ménages (ECAM4) - Tendances, profil et déterminants de la pauvreté au Cameroun entre 2001–2014. 2015)

^c^ Missed prescribed drug doses or not fully respecting the prescription schedule

^d^ Stock-out of at least one of the 3 most prescribed antiretroviral regimens in the previous 12 months: TDF + 3TC + EFV, AZT + 3TC + NVP and TDF + 3TC + NVP. A regimen was considered out of stock if i) the 3-molecule combination was not available and ii) it was not possible to reconstitute the combination using separate single molecules

### History of HBV testing

Only 302 patients (17.7, 95% CI 16.0–19.6) had been tested for HBsAg prior to the study according to data collected from routine medical records and patients’ interviews. Of these, 34 (11.3%) had been found positive. Nine of these 34 patients (26.5%) were HBsAg negative at the time of the present study. Thus, of the 159 patients who were HBsAg positive at the time of the present study, only 25 (15.7%) had a known HBV coinfection prior to the study.

**Fig. 1:**
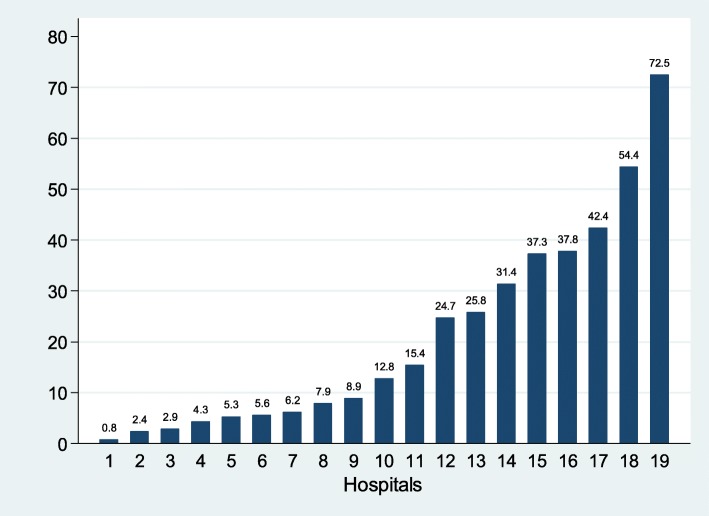
Proportion of patients tested for HBsAg prior to the study, by hospital

HBV testing had been performed prior to ART initiation in 151 patients (50.0%; median time 35 days, IQR 13–151), the day of ART initiation in 21 patients (7%), and after ART initiation in 123 patients (40.7%; median time 42.3 months, IQR 18.7–65.3). The proportion of patients tested for HBsAg ranged from 0.8 to 72.5% according to the individual hospital (*p* < 0.001; Fig. 1).

Factors associated with a history of HBV testing are shown in Table 2. A history of HBV testing was lower in women than in men (15.7% versus 23.2%; adjusted odds ratio [aOR] 0.64, 95% CI 0.46–0.89, *p* = 0.010). By contrast, a history of HBV testing was higher in patients with a secondary or higher educational level than in those with a lower school educational level (20.4% versus 12.8%; aOR 1.38, 95% CI 1.02–1.90, *p* = 0.042), and higher in patients who initiated ART in 2010 or later than in those who started ART prior to 2010 (19.9% versus 14.5%; aOR 1.66, 95% CI 1.23–2.27, *p* < 0.001). Finally, a history of HBV testing was higher in patients with increased ALT level (aOR 1.26 per 10 IU/L increase, 95% CI 1.15–1.39, *p* < 0.001). It is worth noting that a history of HBV testing was not associated with any hospital-related characteristic such as the region, setting and administrative sector of study hospital, type of HIV service, and task-shifting of ART prescription renewals or follow-up consultations to nurses.

**Table 2:** Factors associated with HBV testing in HIV-infected patients using multilevel logistic regressions

	HBsAg tested		Univariate analysis			Multivariate analysis		
	n	(%)	OR	95% CI	*p*	Adjusted OR	95% CI	*p*
Gender								
Men	103	(23.2%)	1.00			1.00		
Women	199	(15.7%)	0.59	0.43–0.79	< 0.001	0.64	0.46–0.89	0.010
School educational level								
Lower than secondary	76	(12.8%)	1.00			1.00		
Secondary or higher	226	(20.4%)	1.53	1.11–2.11	0.010	1.38	1.02–1.90	0.042
Living below poverty line^a^								
No	32	(25.4%)	1.00					
Yes	270	(17.9%)	0.60	0.39–0.97	0.036			
Use of razor blade or clipper of another person								
Never	177	(16.5%)	1.00					
Sometimes	122	(20.2%)	1.44	1.07–1.95	0.017			
Time of ART initiation								
Prior to 2010	101	(14.5%)	1.00			1.00		
2010 or after	201	(19.9%)	1.45	1.12–1.88	0.005	1.66	1.23–2.27	< 0.001
ALT level (per 10 IU/L increase)			1.12	1.06–1.18	< 0.001	1.26	1.15–1.39	< 0.001

Abbreviations: *ART* antiretroviral therapy, *CI* confidence interval, *OR* odds ratio, *ALT* Alanine aminotransferase

^a^ Household monthly income < 28,310 Francs CFA, approximately 43 Euros

The following characteristics were not associated with HBV testing: age; residence setting; marital status; paid activity; household monthly income; number of meals per day; HIV clinical stage at ART initiation; CD4 cell count; HIV viral load; AST level; body mass index; hospitalization in the previous 3 months; consultation of a traditional healer in the previous 3 months; history of incarceration; scarification, tattoo or piercings; number of sexual partners during lifetime; beer consumption; journey time to arrive at study hospital; region, setting and administrative sector of study hospital; type of HIV service; task-shifting of ART prescription renewals or follow-up consultations to nurses

### HBV treatment

Of the 34 patients who were known HBsAg positive prior to the study, 29 (85.3%) received TDF + 3TC (or FTC), three (8.8%) 3TC (or FTC) alone, one (2.9%) TDF alone, while one (2.9%) did not receive any anti-HBV drug. The proportion of patients with TDF + 3TC (or FTC) was significantly higher in those patients who had been found HBsAg positive than in the patients who had been found HBsAg negative or who had not been tested (85.3% versus 59.9%, *p* = 0.002).

Of the 1706 study patients, 1028 (60.5%) received TDF + 3TC (or FTC), 582 (34.2%) 3TC (or FTC) alone, 40 (2.4%) TDF alone, and 50 (2.9%) did not receive any anti-HBV drug (treatment unknown for six patients). Of the 159 patients with HBsAg positive, 97 (61.0%) received TDF + 3TC (or FTC), 56 (35.2%) 3TC (or FTC) alone, three (1.9%) TDF alone, and two (1.3%) did not receive any anti-HBV drug (treatment unknown for one patient).

### HBV suppression

Of the 159 patients with HBsAg positive, 157 had a HBV viral load measurement. Of the latter, 114 had a viral load below 10 IU/mL (72.6, 95% CI 65.1–79.0). Genotyping was successful in 22 of the 27 patients with HBV DNA above 250 IU/mL; 11 were infected with genotype A1 strains and 11 with genotype E strains.

Factors associated with HBV suppression are shown in Table 3. HBV suppression was higher in patients with a secondary or higher educational level than in those with a lower educational level (77.7% versus 63.0%; aOR 2.31, 95% CI 1.05–5.07*, p =* 0.037), higher in patients with a HIV viral load below 300 copies/mL than in those with a viral load above 300 copies/mL (78.3% versus 54.1%; aOR 3.46, 95% CI 1.48–8.09, *p =* 0.004), and higher in patients followed up in a secondary HIV service than in those followed up in a primary HIV service (81.9% versus 64.7%; aOR 2.79, 95% CI 1.24–6.27, *p* = 0.013). By contrast, HBV suppression was lower in patients followed up in the Littoral region than in those followed up in the Centre region (66.7% versus 76.3%; aOR 0.42, 95% CI 0.19–0.96, *p* = 0.039). Finally, HBV suppression was lower in patients with increased ALT level (aOR 0.86 per 10 IU/mL increase, 95% CI 0.75–0.97, *p* = 0.019).

**Table 3:** Factors associated with HBV suppression in HIV/HBV coinfected patients using multilevel logistic regressions

	HBV viral load < 10 IU/mL		Univariate analysis			Multivariate analysis		
	n	(%)	OR	95% CI	*p*	Adjusted OR	95% CI	*p*
School educational level								
Lower than secondary	34	(63.0%)	1.00			1.00		
Secondary or higher	80	(77.7%)	2.04	0.97–4.27	0.058	2.31	1.05–5.07	0.037
CD4 cell count (per 100 cells/mm^3^ increase)			1.19	1.01–1.41	0.033			
HIV viral load (copies/mL)								
≥ 300	20	(54.1%)	1.00			1.00		
< 300	94	(78.3%)	3.26	1.42–7.46	0.005	3.46	1.48–8.09	0.004
ALT level (per 10 IU/L increase)			0.91	0.81–1.02	0.111	0.86	0.75–0.97	0.019
AST level (per 10 IU/L increase)			0.86	0.75–0.99	0.033			
Time since ART initiation (per 5-year increase)			2.44	1.11–5.34	0.025			
Duration of 3TC/FTC monotherapy (per 1-year increase)			1.18	1.01–1.36	0.042			
Adherence to ART in the previous 4 weeks								
Perfect adherence	26	(59.9%)	1.00					
Adherence difficulties^a^	60	(76.9%)	2.40	1.04–5.52	0.039			
Treatment interruption more than 2 consecutive days	28	(80.0%)	2.83	0.97–8.21	0.055			
Region of study hospital								
Centre	74	(76.3%)	1.00					
Littoral	40	(66.7%)	0.58	0.25–1.35	0.206	0.42	0.19–0.96	0.039
Type of HIV service								
Primary	55	(64.7%)	1.00			1.00		
Secondary	59	(81.9%)	2.47	1.17–5.22	0.017	2.79	1.24–6.27	0.013

Abbreviations: *ART* antiretroviral therapy, *CI* confidence interval, *OR* odds ratio, *ALT* Alanine aminotransferase, *AST* Aspartate aminotransferase

^a^ Missed prescribed drug doses or not fully respecting the prescription schedule

The following characteristics were not associated with HBV suppression: gender; age; residence setting; marital status; paid activity; household monthly income; living below poverty line; number of meals per day; body mass index; anti-HBV drugs as part of ART; duration of current ART; hospitalization in the previous 3 months; consultation of a traditional healer in the previous 3 months; journey time to arrive at study hospital; setting and administrative sector of study hospital; task-shifting of ART prescription renewals or follow-up consultations to nurses; stock-out of ART in the previous 12 months

## Discussion

This large multicentre study in Cameroon showed that key elements of the cascade of HBV care – namely testing, treatment, and virologic suppression - were poorly managed in HIV-infected patients, although these latter were followed up in the national antiretroviral programme. As a consequence, a substantial proportion of HIV/HBV coinfected patients had a high risk of liver disease progression. This situation was especially worrying given that this study also confirmed that HIV/HBV coinfection is frequent in the country (9.3%).

One of the main results was indeed that the response to anti-HBV treatment was rather poor, with 27.4% of coinfected patients having an unsuppressed HBV viral load after a median time on ART of approximately 4 years [17]. It is worth noting that elevated HBV viral load is a risk factor for liver cirrhosis and hepatocellular carcinoma [18, 19]. The poor therapeutic response could be related to ART adherence issues. Although this hypothesis was not supported by the analysis of the association between self-reported adherence in the previous 4 weeks and HBV suppression, it was suggested by the concomitant relatively low proportion of coinfected patients with undetectable HIV viral load (76.4%) and the strong positive relationship between HIV viremia and HBV viremia.

In addition, patients with unsuppressed HBV viral load were more likely to have an increased ALT level, suggesting uncontrolled liver inflammation and fibrosis. Increased ALT level is also a risk factor for hepatocellular carcinoma [20]. In a context where HBV DNA level is generally not monitored, HIV viral load and ALT level could provide useful indications for the management of HBV infection.

Although the nationally and internationally recommended treatment of TDF + 3TC (or FTC) was more commonly used as part of ART in the few patients who had known HBV coinfection prior to the study, than in those who had been found HBsAg negative or who had not been tested, 15% of the former did not receive this treatment [6, 21]. Moreover, approximately 40% of all HIV/HBV coinfected patients diagnosed in this study did not receive the recommended treatment because HBV testing in the routine healthcare setting was scarce and because TDF + 3TC (or FTC) in HIV-infected patients was not systematically provided. This association became the preferred choice in Cameroon after the study in 2015.

A history of HBV testing was indeed very uncommon overall (17.7%) but also very heterogeneous across the 19 study hospitals (ranging from 0.8 to 72.5%). These data are in accordance with published data in Africa. In The Gambia, 21.5% of patients followed up in the largest HIV treatment centre had been tested [22]. A multi-country study also reported a proportion of 21.5% overall, with large differences between the countries - between 0.7% (in Kenya) and 96.0% (in South Africa) [8]. Moreover, similarly to our finding, a study in Zambia showed a great heterogeneity between 15 treatment centres [23]. The reasons for such a heterogeneity in our study are unclear and merit further investigations, especially as a history of HBV testing was not associated with available characteristics of study hospitals (but it was probably associated with unmeasured characteristics).

Patients who started ART in 2010 or later were slightly more likely to have been tested for HBV than those who started ART earlier (19.9% versus 14.5%). The increase in testing from 2010 onwards reflects data in other African countries [8, 23] and could be related to the addition of HBsAg testing in the 2010 WHO recommendations [5]. However, our findings suggest that HIV-infected patients not tested for HBV before starting ART were rarely tested subsequently. This sub-population should be tested as soon as possible so that patients diagnosed with HBV can be started on TDF + 3TC (or FTC), maintained on anti-HBV drugs as part of ART (even in case of drug-related side effects or HIV resistance) to avoid HBV flare, monitored for liver disease progression, and educated on prevention of disease progression and HBV transmission [24, 25].

Our figures suggest that women are much more disadvantaged in terms of the possibility to have HBV testing as compared to men (15.7% versus 23.2%). A similar finding has been reported in Zambia [23]. This female vulnerability has two main detrimental effects: poorer management of HBV infection in women, and increased risk of mother-to-child or sexual transmission of HBV [26].

The main strength of this study is that HBV testing, treatment and virologic suppression in HIV-infected patients were investigated in several very different hospital settings (e.g. urban versus rural, primary versus secondary, public versus private).

However, this study has several limitations. First, our study population may not be representative of the whole population followed up in Cameroon’s national antiretroviral programme. Indeed, the study was performed only in the two regions that include the two major cities (Yaoundé and Douala, the political and economic capitals, respectively) and that are the most experienced in HIV care (out of all 10 regions in Cameroon). In addition, although we sought to select a representative sample of hospitals in these two regions and then a representative sample of patients in these hospitals, urban hospitals were over-represented. Lastly, patients who died or were lost to follow-up prior to the study – and were therefore not included in this cross-sectional survey - are less likely to be tested and/or appropriately treated than included patients. As a result, the management of HBV coinfection in people living with HIV in Cameroon could be even poorer than what was observed in this study.

Second, this study was performed in 2014 and TDF + 3TC (or FTC) is now more commonly used in first-line antiretroviral regimens. However, this study showed that it is not enough to provide TDF-based ART to ensure good HBV care (28.7% of coinfected patients on TDF + XTC had unsuppressed HBV viral load). HBV testing remains crucial for the management of HBV infection (e.g. monitoring of HBV-related complications and choice of second- or third-line ART). The decision of some AIDS programmes (including Cameroon’s since 2016) not to test for HBV because TDF + 3TC (or FTC) is included in standard antiretroviral regimens should be reviewed. In addition, HBV DNA level is not monitored in the routine healthcare setting and data are useful to inform programmes and minimise the risk of liver disease progression. Finally, this study provides baseline data to assess progress towards the 2030 elimination targets.

Third, HBV coinfections could have been missed as HBsAg-negative samples were not further tested. On the one hand, occult hepatitis B can arise especially in HIV-infected patients [27, 28]. On the other hand, HBsAg may have been lost in a substantial proportion of patients as most of them had received ART for several years prior to the study [29, 30]. For instance, 26.5% of the patients who had been found HBsAg positive prior to the study were HBsAg negative at the time of the study (although false results may not be excluded). HBsAg loss on ART may have led to an under-estimation of HBV suppression as patients who lose their HBsAg are always HBV suppressed.

## Conclusion

Improving the management of HBV infection in the routine healthcare setting in Africa is urgently required in order to achieve the 2030 elimination targets. Micro-elimination of HBV infection in people living with HIV who are at higher risk of liver disease progression could be an easier and cost-effective component than more widely scaling up HBV policies, thanks to the availability of TDF + 3TC (or FTC) and follow up of patients in the AIDS programmes. Collaboration between AIDS and hepatitis programmes will be crucial to address the challenge.

## Data Availability

Due to French law there are restrictions on publicly sharing the data of this study. French law requires that everyone who wishes to access cohort data or clinical study data on humans must make a request to the French Data Protection Authority (Commission Nationale de l’Informatique et des Libertés - CNIL), by filling in a form which can be provided by Christian Laurent at the IRD (christian.laurent@ird.fr). For further information, please see: https://www.cnil.fr/.

## Abbreviations

3TC: Lamivudine
ALT: Alanine aminotransferase
ART: Antiretroviral therapy
AST: Aspartate aminotransferase
BIC: Bayesian Information Criterion
CI: Confidence interval
DNA: Deoxyribonucleic acid
FTC: Emtricitabine
HBsAg: Hepatitis B surface antigen
HBV: Hepatitis B virus
HIV: Human immunodeficiency virus
IQR: Interquartile range
OR: Odds ratio
RNA: Ribonucleic acid
TDF: Tenofovir disoproxil fumarate
WHO: World Health Organization
